# SiC Electrochemical Sensor Validation for Alzheimer Aβ_42_ Antigen Detection

**DOI:** 10.3390/mi14061262

**Published:** 2023-06-16

**Authors:** Brayan Montero-Arevalo, Bianca I. Seufert, Mohammad S. Hossain, Evans Bernardin, Arash Takshi, Stephen E. Saddow, Norelli Schettini

**Affiliations:** 1 Department of Electrical and Electronic Engineering, Universidad del Norte, Barranquilla 081007, Colombia; 2 Department of Electrical Engineering, University of South Florida, Tampa, FL 33620, USA; bseufert@usf.edu (B.I.S.); mh666@usf.edu (M.S.H.); ebernardin@usf.edu (E.B.); atakshi@usf.edu (A.T.); saddow@usf.edu (S.E.S.)

**Keywords:** Alzheimer sensor, Aβ-42 detection, electrochemical sensor, biosensor, Silicon-Carbide (SiC) electrode, gold electrode, Cyclic Voltammetry (CV), Electrochemical Impedance Spectroscopy (EIS)

## Abstract

Alzheimer’s disease (AD) is a neurodegenerative disease with only late-stage detection; thus, diagnosis is made when it is no longer possible to treat the disease, only its symptoms. Consequently, this often leads to caregivers who are the patient’s relatives, which adversely impacts the workforce along with severely diminishing the quality of life for all involved. It is, therefore, highly desirable to develop a fast, effective and reliable sensor to enable early-stage detection in an attempt to reverse disease progression. This research validates the detection of amyloid-beta 42 (Aβ_42_) using a Silicon Carbide (SiC) electrode, a fact that is unprecedented in the literature. Aβ_42_ is considered a reliable biomarker for AD detection, as reported in previous studies. To validate the detection with a SiC-based electrochemical sensor, a gold (Au) electrode-based electrochemical sensor was used as a control. The same cleaning, functionalization and Aβ_1–28_ antibody immobilization steps were used on both electrodes. Sensor validation was carried out by means of Cyclic Voltammetry (CV) and Electrochemical Impedance Spectroscopy (EIS) aiming to detect an 0.5 µg·mL^−1^ Aβ_42_ concentration in 0.1 M buffer solution as a proof of concept. A repeatable peak directly related to the presence of Aβ_42_ was observed, indicating that a fast SiC-based electrochemical sensor was constructed and may prove to be a useful approach for the early detection of AD.

## 1. Introduction

Dementia, an early sign of Alzheimer’s disease, is a syndrome in which cognitive deterioration occurs and usually affects the elderly [1]. Currently, this disease affects around 55 million people around the world, and according to the world health organization, there are 10 million new cases reported each year. Alzheimer’s disease (AD) accounts for 60 to 70 percent of dementia cases and has no known cure, although early detection does allow for treatment to slow its progression [1].

AD is a type of brain disease caused by damage to the nerve cells in the brain, and the biggest challenge with this disease is that the first set of symptoms normally does not appear during the earliest stages of the disease. Symptoms may take 20 years or more to appear and are already a reflection of advanced brain damage. AD, when diagnosed, results in a life expectancy of 4 to 7 years [2]. One of the most accepted hypotheses, still under study, about the origin of AD is the so-called Amyloid cascade hypothesis [3]. This hypothesis states that Alzheimer’s disease is caused by an abnormal accumulation of Amyloid Beta (Aβ) plaques in various areas of the brain. These plaques act as a trigger for a cascade effect that includes neuronal injury and the formation of neurofibrillary tangles via the tau protein, which in turn leads to neuronal dysfunction and cell death for patients with AD [4,5]. The origin of AD can be traced back to Aβ peptides, which are present in two lengths of amino acids, Aβ_40_ and Aβ_42_, containing 40 and 42 amino acids, respectively. Aβ is produced through enzymatic action on the amyloid precursor protein (APP) by β and γ-secretase. This, in turn, releases the Aβ peptides, including Aβ_40_ and Aβ_42_ monomers, with the latter considered as the pathogenic species [4,5]. Consequently, Aβ_42_ monomers experience conformational change, first assembling into Aβ_42_ oligomers and then a continuous aggregation into Aβ_42_ fibers. These fibers then accumulate in the brain as amyloid plaques. Recent studies conducted by Castellani et al. also confirm the predominance of Aβ_42_ in affected areas of diagnosed AD brain tissue [5].

The available diagnostic measures for Alzheimer’s include psychological, genetic, brain imaging, and cerebrospinal fluid (CSF) tests [6]. Psychological tests are quite inaccurate, and genetic tests are not always available. Brain imaging can be expensive, while CSF tests are invasive and extremely painful for the patient [7,8]. Considering that the treatment for AD can be more effective if it starts at the early stages of the disease, a simple, reliable, and relatively inexpensive early-stage diagnostic method should prove to be a tremendous improvement.

The reported studies on antibody-based biosensors are shown in Table 1, where it is observed that certain characteristics predominate, such as gold and carbon electrode materials and the use of mouse monoclonal antibodies. It is important to note that Lien et al. used a human monoclonal antibody [9], Rama et al. and Dai et al. used a rabbit monoclonal antibody [10,11], and Li et al. and Hsu et al. used a rabbit polyclonal antibody [12,13]. The latter draw attention because they are more tolerant to small changes in the nature of the antigen and are reported to offer more robust detection [14]. In practice, an AD biosensor would require a liquid sample for operation, which in most cases is CSF, which requires an invasive lumbar puncture. Non-invasive attempts have been made targeting other bodily fluids, such as blood and saliva. However, in these bodily fluids, the Aβ concentration is lower than in the CSF, where the cut-off value to differentiate between patients with dementia and healthy patients is 500 pg·mL^−1^. This requires detection techniques that allow for a significantly lower limit of detection (LOD) [15].

As observed in Table 1, most of the reported electrochemical biosensors have a LOD below the cut-off value to differentiate patients with dementia; this is due to their advantages regarding selectivity, sensitivity, and response time compared to other methods [16]. Among the parameters that can be measured with antibody-based biosensors, the following are the most common:

- Electrical Current: Used in techniques such as Cyclic Voltammetry (CV), Square Wave Voltammetry (SWV), Differential Pulse Voltammetry (DPV), and Chronoamperometry (CA).
- Electrical Impedance: Used in Electrochemical Impedance Spectroscopy (EIS).
- Optical Luminescence: Used in Electrochemiluminescence (ECL).

Throughout the years, electrochemical biosensors have been used for biomedical research and many medical applications, mainly due to their simplicity, affordability, point-of-care strategies [17] and, in many cases, better LOD than other methods [16]. Additionally, antibodies are suited for element biorecognition because they provide the sensor with high specificity and sensitivity [18]. The application of metallic or carbon-based electrodes limits the potential window of an electrochemical-based biosensor to 1.23 V [19], at which water electrolysis occurs. However, wide energy bandgap materials, such as 4H-SiC, which has a potential window of 3.2 V, allow for the targeting of a wider range of organic molecules [20].

Thanks to its wide energy bandgap, silicon carbide (SiC) is characterized by low leakage currents and very low electronic noise [21], which makes it suitable as an electrode for biosensors and neuro-implant applications [22]. Furthermore, its performance in harsh environments [23] makes it ideal for the development of reusable biosensors since it can be subjected to multiple chemical processes using etching techniques without suffering any deterioration in its chemical properties.

**Table 1:** Electrochemical biosensors based on antibodies for Aβ_42_ detection.

Method *	Electrode Material	Functionalizing Antibody	Aβ_42_ Solution Media	Aβ_42_ Detection Range **	Aβ_42_ LOD **	Refs.
CV	Gold electrode	Aβ_1–42_	PBS	100–300 μM	100 μM	[24]
SWV	Carbon fiber microelectrode	mHJ2, mHJ7.4	Mice CSF	20–140 nM	20 nM	[25]
CV	Gold electrode	6E10	aCSF	0.02–1.50 nM	10 pM	[26]
EIS	AAO Sensing electrode	12F4	BSA	$1–10,000\;\mathrm{pg}\cdot {\mathrm{mL}}^{-1}$	$1\;\mathrm{pg}\cdot {\mathrm{mL}}^{-1}$	[27]
CV	Screen printed carbon electrode	H31L21	BSA	$0.5–500\;\mathrm{ng}\cdot {\mathrm{mL}}^{-1}$	$0.1\;\mathrm{ng}\cdot {\mathrm{mL}}^{-1}$	[10]
CV	Gold electrode	6E10 12F4	aCSF	0.5–50 nM 0.05–0.5 nM	5 pM	[28]
CV, EIS	Gold Electrode	6E10	Nutrient Mixture F12	-	~5 pM	[29]
EIS	Carbon printed electrode	antimAβ	0.02% (*v*/*v*) ammonia water at 200 mM concentration	0.01–100 nM	0.57 nM	[9]
CA	Screen printed carbon electrode	anti-Aβ	Human CSF, Serum and Plasma	$20–12500\;\mathrm{pg}\cdot {\mathrm{mL}}^{-1}$	$19\;\mathrm{pg}\cdot {\mathrm{mL}}^{-1}$	[30]
CV, DPV	Screen printed gold electrode	Aβ_1–28_	CSF	$5–800\;\mathrm{pg}\cdot {\mathrm{mL}}^{-1}$	$5\;\mathrm{pg}\cdot {\mathrm{mL}}^{-1}$	[12]
SWV, EIS	Gold electrode	DE2B4	PBS	$10–1000\;\mathrm{pg}\cdot {\mathrm{mL}}^{-1}$	$5.2\;\mathrm{pg}\cdot {\mathrm{mL}}^{-1}$	[31]
ECL	Glassy Carbon Electrode	anti-Aβ	PBS	$80\;\mathrm{fg}\cdot {\mathrm{mL}}^{-1}–100\;\mathrm{ng}\cdot {\mathrm{mL}}^{-1}$	$52\;\mathrm{fg}\cdot {\mathrm{mL}}^{-1}$	[32]
SWV, CV, EIS	Glassy Carbon Electrode	anti-Aβ	PBS	$0.0001–100\;\mathrm{ng}\cdot {\mathrm{mL}}^{-1}$	$0.03\;\mathrm{pg}\cdot {\mathrm{mL}}^{-1}$	[33]
CV	Screen printed carbon electrode	12F4, 1E11	Human Serum and Plasma	100 fM–25 nM	100 fM	[34]
DPV, EIS	Gold Electrode	EPR9296	PBS and Human Serum	$0.0675–0.5{\;\mu g\;\mathrm{mL}}^{-1}$	$0.0675\;\mu g\cdot {\mathrm{mL}}^{-1}$	[11]
LSV	Conductive silk fibroin-based immunoparticles	mOC31	Serum	$22.5–1125\;\mathrm{pg}\cdot {\mathrm{mL}}^{-1}$	$3.74\;\mathrm{pg}\cdot {\mathrm{mL}}^{-1}$	[35]
EIS, CV	ICE (Ti+Au)	anti-Aβ	Human Serum	$0.01–10,000\;\mathrm{ng}\cdot {\mathrm{mL}}^{-1}$	$100\;\mathrm{pg}\cdot {\mathrm{mL}}^{-1}$	[36]
EIS	ICE	anti-Aβ	Human Serum	$10–100,000\;\mathrm{pg}\cdot {\mathrm{mL}}^{-1}$	$7.5\;\mathrm{pg}\cdot {\mathrm{mL}}^{-1}$	[37]
DPV	Graphene-modified Screen-printed electrode	H31L21	Spiked human and mice plasmas	11 pM–55 nM	2.398 pM	[38]
EIS	Gold Electrode	12F4	PBS	$10{\;\mathrm{pg}\;\mathrm{mL}}^{-1}–100{\;\mathrm{ng}\;\mathrm{mL}}^{-1}$	$113{\;\mathrm{fg}\;\mathrm{mL}}^{-1}$	[39]
CV	Gold Electrode	Aβ_1–28_	PBS	$0.1{\;\mathrm{pg}\;\mathrm{mL}}^{-1}–10,000{\;\mathrm{ng}\;\mathrm{mL}}^{-1}$	$10.4{\;\mathrm{fg}\;\mathrm{mL}}^{-1}$	[13]

* LOD: Limit of Detection, CV: Cyclic Voltammetry, DPV: Differential Pulse Voltammetry, LSV: Linear Sweep Voltammetry, SWV: Square Wave Voltammetry, AAO: Anodic Aluminum Oxide, mAb: Monoclonal Antibody, aCSF: Artificial Cerebrospinal Fluid, ECL: Electrochemiluminescence, ICE: Interdigitated chain-shaped electrode. BSA: Bovine Serum Albumin. ** The units are reported as they appear in their respective research citations.

This work compares the electrochemical performance of bare gold (Au) and hexagonal crystalline silicon carbide (4H-SiC) electrodes [40] for Aβ_42_ detection. The presented results here show distinct features in the 4H-SiC electrode due to the feasibility of testing the sensor at a larger voltage range than what can be applied to a gold electrode.

## 2. Experimental

### 2.1. Apparatus and Electrodes

The CV and EIS measurements were carried out using a VersaSTAT 4 Potentiostat, where the 4H-SiC and Au electrodes were incorporated into a conventional three-electrode cell configuration. The 4H-SiC and Au electrodes were developed in previous works [40,41], respectively, then refurbished and used in this preliminary study.

**Figure 1:**
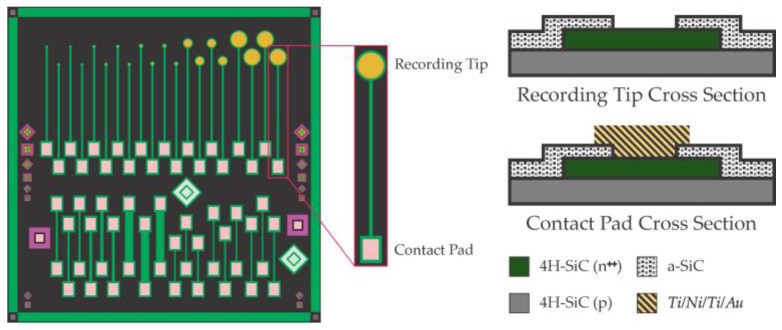
Single-ended electrodes (**top left image**) with various recording areas (diameters of 25, 50, 100, 400, and 800 μm) and test structures. The right shows the device’s cross-section construction at both the recording tip (**top right**) and the metal contact pad (**bottom right**).

The fabrication and performance of the 4H-SiC electrodes were detailed in Bernardin et al. [42]; however, the experimental procedure used in this work has been refined as it follows [40]. Figure 1 shows the mask design used to produce the single-ended 4H-SiC electrodes (top) and the test structures (bottom), along with the device cross-section delineating all materials used to construct the sensor.

4H-SiC sensor fabrication started with an epiwafer consisting of a p base layer (doping ~ 1 × 10^16^ cm^−3^) capped with a heavily doped n^+^ layer (doping ~ 5 × 10^18^ cm^−3^). The electrodes were realized using various standard microelectronic fabrication methods resulting in the device cross-section shown in Figure 1. The novel aspect of this device was the use of a degenerately doped n^+^ 4H-SiC electrode layer to realize metallic-like electron transport in a semiconductor mesa. Amorphous SiC (*a*-SiC) was used as a conformal insulator, thus resulting in a low-electrical impedance device with only SiC materials in contact with the analyte [43] and, in this case, the electrochemical environment.

**Figure 2:**
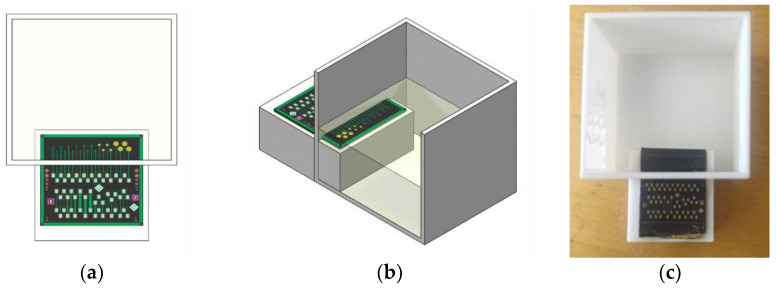
4H-SiC electrode schematic showing (**a**) a top view of the recording tip region submerged in the electrochemical solution and (**b**) a 3D view of the 4H-SiC electrodes packaged in a liquid containment well. (**c**) Photograph of the 4H-SiC electrode mounted in the test well (white).

The 4H-SiC electrodes were packaged in an electrochemical test well 3D printed in PLA for this purpose, as shown in Figure 2b, whereby the recording tips were exposed to the electrochemical solution while the rest of the sensor die (i.e., bonding pads) were dry (Figure 2a), thus allowing for connection of the metal bonding pads to the electrochemical apparatus.

**Figure 3:**
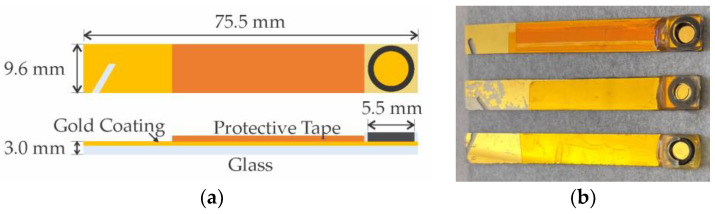
(**a**) Top and side view diagram of the gold electrodes. (**b**) Photograph of the 75.5 mm long, 9.6 mm wide electrodes.

The Au electrodes (RMS roughness < 2 nm) as shown in Figure 3b, were fabricated through the evaporation of an adhesion layer of 20 nm of chromium at a deposition rate of 2 Å·s^−1^ followed by 500 nm of gold at a deposition rate of ~1 Å·s^−1^ onto glass substrates [41].

### 2.2. Reagents and Solutions

Phosphate buffer solution (PBS), 3-Mercaptopropionic acid (MPA), *N*-(3-Dimethylaminopropyl)-*N*′-ethylcarbodiimide hydrochloride (EDC), *N*-hydroxysuccinimide (NHS), concentrated sulfuric acid (H_2_SO_4_ 95.0 to 98.0 *w*/*w*%), concentrated nitric acid (HNO_3_ 70% *w*/*w*%), K_3_[Fe(CN)_6_], K_4_[Fe(CN)_6_], sodium hydroxide (NaOH) and dimethyl sulfoxide (DMSO) were purchased from Sigma Aldrich and used in this study as described below.

### 2.3. Antibody and Antigen Solutions

1 mg of IgG in 0.1 mL (1 mg·mL^−1^) of PBS pH 7.4 with 0.09% sodium azide Amyloid Beta [1,2,3,4,5,6,7,8,9,10,11,12,24,25,26,27,28,29,30,31,32,33,34,35,36,37,38,39] rabbit polyclonal antibody and 1 mg of lyophilized solid packaged Amyloid Beta [1,2,3,4,5,6,7,8,9,10,11,12,13,14,15,16,17,18,19,20,21,22,23,24,25,26,27,28,29,30,31,32,33,34,35,36,37,38,39,40,41,42] Peptide were obtained from Abbiotec. For the dissolution of the Amyloid Beta [1,2,3,4,5,6,7,8,9,10,11,12,13,14,15,16,17,18,19,20,21,22,23,24,25,26,27,28,29,30,31,32,33,34,35,36,37,38,39,40,41,42] Peptide, Abbiotec recommends the use of 100% DMSO, and for Amyloid Beta [1,2,3,4,5,6,7,8,9,10,11,12,24,25,26,27,28,29,30,31,32,33,34,35,36,37,38,39] antibody, 0.1 M PBS solution was added until a concentration of 18.75 µg·mL^−1^ was reached.

This antibody was selected because it is a highly specific antibody [13], and it has also been used successfully on gold electrodes [12,13]. The Aβ concentration in solution during the functionalization of the electrode surface is crucial for successful detection. If the concentration is high while the Aβ_42_ target concentration is low, the electrochemical reaction will likely not be detected. For this reason, both the antibody and antigen concentrations of 18.75 μg·mL^−1^ and 0.5 μg·mL^−1^, respectively, were taken from Dai et al. research, where Aβ_42_ detection was successfully reported using a gold electrode [11].

### 2.4. Methods

#### 2.4.1. Cleaning

**Figure 4:**
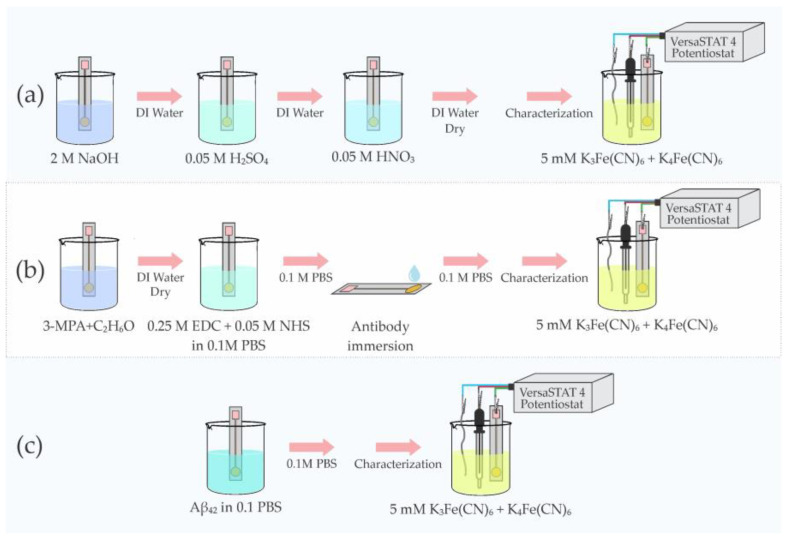
Electrode preparation procedure (**a**) Cleaning, (**b**) Functionalization and antibody immobilization, and (**c**) Antigen Detection.

Before starting the electrochemical experiments, the electrodes were cleaned based on the studies carried out previously [11,44,45]. The electrodes were first immersed in a 2 M NaOH solution for 15 min according to the study carried out by Schneider et al. [46] and then rinsed with deionized (DI) water for 30 s. Next, the electrodes were immersed in a 0.05 M H_2_SO_4_ solution for 3–5 min and then rinsed with DI water for 30 s. Lastly, the electrodes were immersed in a 0.05 M HNO_3_ solution for 3–5 min and then rinsed in DI water for 30 s. After the cleaning was completed, the electrodes were left to air dry. Refer to Figure 4a for reference.

#### 2.4.2. Electrochemical Cells

In order to properly and accurately test each electrode, a 3-probe configuration was used for the electrochemical cell setup with an Ag/AgCl reference electrode, Pt wire as the counter electrode, and our electrode under test (Au or 4H-SiC) as the working electrode. All electrodes were then submerged in the liquid electrolyte with a pH of 7.4 with the following chemistry: 0.18417 g of K_4_Fe(CN)_6_, 0.1646 g of K_3_Fe(CN)_6_, and 100 mL PBS.

#### 2.4.3. Procedure & Measurements

Once the electrodes were appropriately cleaned, they were characterized with CV and EIS measurements, as shown in Figure 4a. These readings were designated as the baseline for each electrode’s response. CV measurement settings were as follows: for the Au electrode, the sweep voltage was from −0.5 V to 0.5 V, while for the case of 4H-SiC with a wider electrochemical window, the voltage sweep was from −1.98 to 2.77 V. For EIS measurements a 50 mV amplitude signal with a frequency range from 10 kHz to 0.1 Hz was applied to both electrodes. Upon establishing the baseline performance of each electrode, the electrodes were subsequently functionalized using the procedure shown in Figure 4.

To functionalize the electrodes, they were first immersed in a 1 mM solution of 3-MPA in ethanol for 24 h to achieve a self-assembled monolayer (3-MPA-SAM), rinsed with DI water and then dried gently with air at room temperature. Afterward, the exposed end of the 3-MPA-SAM was functionalized by treating the electrodes in a solution of 0.1 M PBS containing 0.25 M EDC and 0.05 M NHS for 5 h, rinsed with DI water and then dried gently with air at room temperature. Next, the electrodes were immersed in 20 mL of Aβ antibody solution (Aβ_1–28_) with a concentration of 18.75 µg·mL^−1^ for 20 h at 4 °C, rinsed with 0.1 M PBS and stored at 4 °C. Once functionalized, CV and EIS measurements with the sensors were performed to validate their response to antibody exposure.

After the antibody response measurements were completed, a solution of Aβ_42_ Antigen dissolved with DMSO to prevent aggregation was diluted with 0.1 M PBS solution to reach an Aβ_42_ concentration of 0.5 μg·mL^−1^. The diluted solution was incubated for 30 min at room temperature on the top of each biosensor to avoid aggregation of Aβ_42_ during incubation [11]. After the incubation, the biosensor was rinsed in 0.1 M PBS, and CV and EIS measurements of the sensor response to the Aβ_42_ Antigen were then made. CV and EIS measurements were then performed in the electrolyte solution described earlier (10 mL solution of K_4_Fe(CN)_6_ and K_3_Fe(CN)_6_ of 5 mM in 0.1 M PBS).

## 3. Results

**Figure 5:**
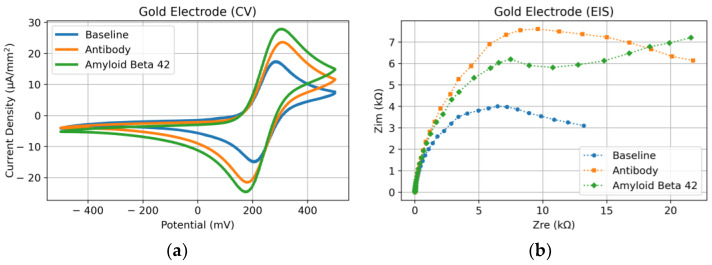
Gold (Au) electrode response after each electrochemical sensor functionalization step is shown in Figure 4. (**a**) CV response and (**b**) EIS response.

**Figure 6:**
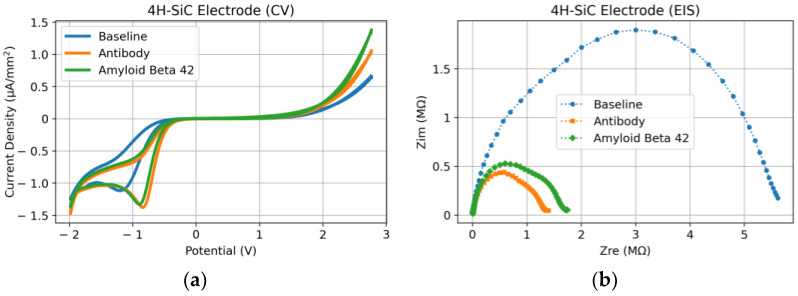
4H-SiC electrode response after each electrochemical sensor functionalization step. (**a**) CV response and (**b**) EIS response.

For clarity, it is best to break the following electrochemical data graphs into a comparison of “steps” that allow for a conclusive analysis of the electrochemical sensor performance (please see the three steps in Figure 4 for reference). In the last section, the device steps are described as baseline (after cleaning), antibody functionalization, and the Aβ_42_ functionalization steps. After each step, the sensors were tested in the aforementioned electrochemical solution and setup. Figure 5 shows the data obtained for the Au electrode, and Figure 6 the data for the 4H-SiC electrode.

As seen in Figure 5a, the CV baseline in the gold electrode shows a reversible reaction with the reduction and oxidation peaks at 290 mV and 210 mV, respectively. After the immobilization of the antibody, the peaks were shifted to 310 mV and 180 mV, and the magnitude of the current was increased. The CV results also show a higher current level after the introduction to Aβ (Aβ_1–28_) antibody. Still, the shape of the curves shows a fully reversible redox reaction on the gold electrode. The EIS results in Figure 5b reveal more details about the differences between the tested samples. The radius of the semicircles at the higher frequencies increased from the baseline to the antibody sample, indicating larger charge transfer resistance in the electrode with the immobilized antibody. While a slight decrease in the radius was observed after the incubation of the antibody, the tail of the curve at lower frequencies shows a distinct feature. As seen in the Nyquist plot, the tail of the impedance at low frequencies represents the mass transfer limitations. It is clear that in the presence of Aβ_1–28_, the mechanism of redox reaction in potassium ferricyanide is more affected by the interaction of the antibody to the immobilized antigen.

The experimental results from the 4H-SiC electrode show a totally different response (Figure 6). First, despite the wider voltage range in the CV experiment, there are only oxidation peaks at negative voltages and no reduction peak. However, the exponential increase in the current at the positive voltages represents the Butler–Volmer model (limitation by the kinetics of the reaction) [47]. Such a rectifying response is expected from a wideband gap semiconductor as the electron donation rate is significantly lower than the electron acceptance rate by the electrode. In addition to the domination of the oxidation process in the SiC electrode, immobilization of the antibody had a significant impact on the oxidation peak, shifting it from −1.19 V in the baseline to −0.85 V in the electrode with the immobilized antibody. However, the peak voltage did not change after the incubation of Aβ_1–28_. Unlike the gold electrode, the EIS results from the 4H-SiC electrode (Figure 6b) show the domination of the semicircle response and almost no tails at the lower frequencies. This again shows that due to the semiconducting property of the electrode, the kinetic rate of the reaction dominates the response of the sensor. While the baseline shows a relatively large semicircle, after adding the antibody, the radius decreased significantly, implying a faster kinetic rate. However, the antigen had a reverse response resulting in a slightly larger semicircle radius (slower reaction rate).

Comparing the two electrodes, the most important observation was that Aβ_42_ was detected by both the Au and SiC sensors, albeit at different voltages and with different amplitudes. It appears that the gold electrode was sensitive enough to utilize a lower voltage input and provide a clearly measurable response compared with the SiC sensor. It should be noted that the contact surface of the 4H-SiC electrode had a diameter of 800 μm compared to 5.5 mm in the case of gold. While the CV results are reported based on the current density, a much smaller electrode surface area in the SiC electrode resulted in a much larger impedance (MΩ range) than that in Au (kΩ range). Nevertheless, incubation of the biomaterials had the opposite effect on the impedance of the gold and SiC by increasing the impedance in the gold electrode and decreasing it in the SiC.

## 4. Conclusions and Future Implications

In conclusion, this paper presents preliminary sensor response data from two electrodes made using Au and SiC, respectively. Both sensors were functionalized and tested for their suitability as effective electrochemical biosensors for Alzheimer’s Antigen (Aβ_42_) detection. The results presented here demonstrate that the Antigen and both sensors resulted in the positive detection of Aβ_42_, a highly important finding. The gold surface resulted in a larger signal response compared to 4H-SiC. It should be noted that the data are very preliminary, and SiC microelectrode array electrodes were used for these tests, which were not of the same form fit as the gold electrodes.

Most importantly, this research contributes to the science of defining a possible diagnostic measurement of the Alzheimer’s Aβ_42_ Antigen. In addition to identical form-fit sensors, future work in this area by our research team will involve the use of other metal oxides to define a baseline of the electrochemical biosensor’s response over time so as to identify the best candidate material for further development of AD diagnostic tools.

## Data Availability

The data presented in this study are openly available in Zenodo at https://doi.org/10.5281/zenodo.8040514.
